# First Report of Human Hepatitis B Virus (HBV) in Wild Neotropical Primates

**DOI:** 10.1007/s10393-026-01787-5

**Published:** 2026-04-03

**Authors:** Jean P. Boubli, Hani Rocha El Bizri, Luan F. Botelho-Souza, Chrysoula Gubili, Stephen J. Martin, Maisa da S. Araújo, Tommy C. Burch, Mariluce R. Messias, Alcione de O. dos Santos, Luiz S. Ozaki, André V. C. Pereira, Tony H. Katsuragawa, Ana Maísa Passos-Silva, Luiz H. S. Gil, Izeni P. Farias, Juan M. V. Salcedo, Deusilene Vieira

**Affiliations:** 1 School of Science, Engineering and the Environment, University of Salford, Salford, Greater Manchester UK; 2 Center for International Forestry Research (CIFOR), Bogor, Indonesia; 3 Laboratório de Virologia Molecular, Fundação Oswaldo Cruz Rondônia - FIOCRUZ/RO, Porto Velho, Rondônia Brazil; 4 Fundação Universidade Federal de Rondônia, UNIR, Porto Velho, Rondônia Brazil; 5 Virginia Commonwealth University, Richmond, VA USA; 6 Instituto Nacional de Ciência e Tecnologia de Epidemiologia da Amazônia Ocidental – INCT-EpiAmO, Porto Velho, Rondônia Brazil; 7 Laboratório de Evolução e Genética Animal, Universidade Federal do Amazonas, Manaus, Amazonas Brazil

**Keywords:** HBV, zoonosis, emerging infectious diseases, one health, biodiversity, deforestation

## Abstract

Globalization and environmental change are reshaping infectious disease dynamics, including reverse zoonotic transmission from humans to wildlife. We assessed hepatitis B virus (HBV) infection in wild New World primates from two Brazilian Amazon regions: an anthropogenically impacted area in southwestern Amazonia (Rondonia and Mato Grosso states) and a remote, minimally impacted area along the upper Japurá River (Amazonas state). Blood or liver samples from 88 primates representing 28 species were examined for HBV. HBV was detected in 17 of 49 primates (34.7%) from disturbed areas but in none of the 39 primates from the remote site. Human population density significantly predicted infection, and viral genotypes matched locally circulating human HBV strains, supporting human-to-primate spillover driven by deforestation and encroachment.

**Supplementary Information:**

The online version contains supplementary material available at https://doi.org/10.1007/s10393-026-01787-5.

Globalization has significantly accelerated the emergence and spread of infectious diseases, particularly those caused by viral pathogens (Smith et al., 2014). Key examples include AIDS, SARS, COVID-19, the Nipah and the Influenza A (H1N1) virus diseases, which have all undergone zoonotic spillovers. While most known cases involve transmission from animals to humans, reverse zoonotic (anthroponotic) transmission from humans to animals has also been documented (Al Noman et al. 2024), such as in polio (Flexner & Lewis, 1910) and human-to-ape hepatitis B virus (HBV) transmission (Köndgen et al., 2010). In fact, anthroponotic transmission from humans to apes has become a great concern for the conservation of these endangered primates (Koster et al*.*, 2022).

Currently, two billion people are infected with HBV, causing nearly one million deaths annually (ICTVdB, 2006). It has been proposed that HBV first appeared in humans around 40,000 years ago via spillover from an unknown non-primate source (e.g. a rodent or bird), before jumping from humans to apes via independent transmission events involving chimpanzees, gibbons, and orangutans (Paraskevis et al., 2013). Human HBV is currently classified into eight distinct genotypes (A to H) (World Health Organization, 2009), and the great and lesser apes (chimpanzees, gorillas, orangutans, and gibbons) carry a group of distinctive nonhuman HBV genotypes (Franchini et al., 1987; Lanford et al., 2000; MacDonald et al., 2000; Heckel et al., 2001; Starkman et al., 2003; Makuwa et al., 2005).

New World primates have inhabited the Americas since the Oligocene, ~ 35 Ma (Schrago and Russo, 2003), but humans arrived in the Americas approximately 20,000 years ago (Paraskevis et al., 2013), bringing with them the HBV-F genotype, which remains restricted to the Americas (Paraskevis et al., 2013). A recent survey in the southwestern Amazon, an area undergoing large-scale deforestation, found that the HBV-A and -D genotypes dominate due to European and African immigration (Santos et al., 2010; Ferreira-Junior et al., 2020). In this region, people have very close relationships with wild monkeys due to hunting and the pet trade (Parathian and Maldonado, 2010; Pereira et al. 2019; Antunes et al. 2025). Hence, HBV may have crossed over to wild nonhuman primates in regions where human occupation of the Amazon forest was occurring.

To test this hypothesis, between July 2009 and November 2013, wild primates were sampled at two Brazilian Amazon sites: a human-impacted region in southwestern Amazonia (Rondônia, Mato Grosso and Amazonas; 49 individuals from 17 species), where the prevalence of hepatitis B virus (HBV) infection is the highest in the country (Ferreira-Junior et al. 2020), and a remote area deep in the Amazon that is mostly unaffected by humans along the upper Japurá River (Amazonas; 39 individuals from 11 species), using blood or liver tissues. (Fig. 1) (Table S1). Details of the sampling procedures and protocols are described elsewhere (Araújo et al., 2013, Supplementary Materials). Viral DNA was extracted from these samples and analysed using polymerase chain reaction (PCR) techniques (Chomczynski, 1993; Sambrook et al., 2006; Tran et al., 2006) (Supplementary materials). Phylogenetic analysis of a 547-bp fragment of the S gene was performed with Bayesian methods (MrBayes 3.2) (Ronquist et al., 2012) (full methodology provided in Supplementary materials).

**Figure 1:**
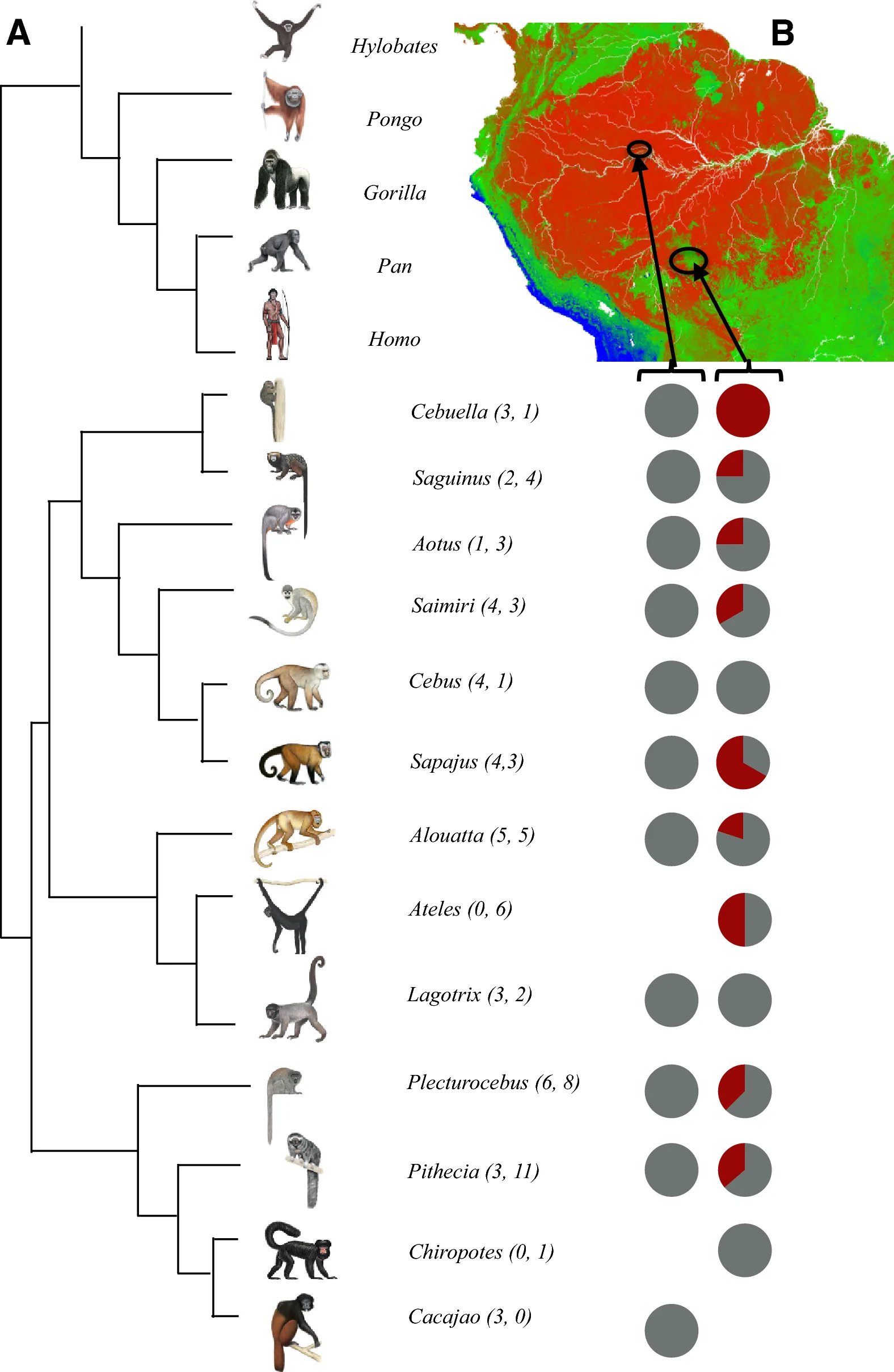
**A** Phylogenetic relationships among all the primate genera considered in this study based on that of Perelman et al. (2011). **B.** Map of the northern region of South America showing the locations where the samples were collected. Red represents dense Amazonian forest and green denotes open areas, including areas of anthropogenic deforestation (source: NASA Observatory). The pie charts show the percentages of sampled primates that tested positive for HBV in the remote Japura River area (left) and Southwestern Amazon (right). Grey denotes negative PCR reactions, and red indicates positive PCR reactions. Each group of pie charts represents individuals in the corresponding primate genus shown on the left in part **A.**

None of the 39 liver tissues from 11 species of monkeys collected from the remote upper Japurá River site tested positive for HBV. However, 17 (34.7%) blood samples from primates collected in the anthropogenically impacted site in southwestern Amazonia tested positive for HBV (Fig. 1) (Table S1). A generalized linear mixed model (GLMM) analysis found that human population density was a significant predictor of HBV infection in primates (Coefficient: 0.13; t-value: 2.11; p-value: 0.039) (Fig. 2). However, methodological explanations for this pattern cannot be excluded, as sample types and analytical approaches differed among sampling locations. Although liver tissue and the methods used are generally considered adequate based on existing literature (Ghosh et al. 2015), this uncertainty should be acknowledged as a potential limitation of the study.

**Figure 2:**
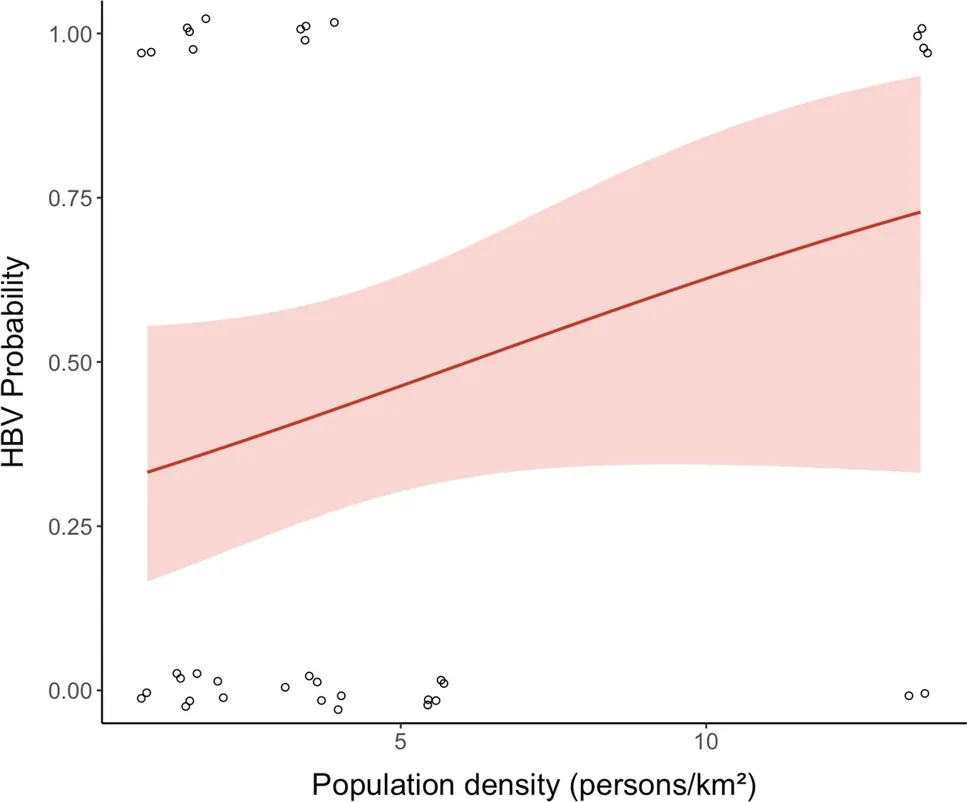
A scatter plot, based on a generalized linear mixed model, showing the positive correlation between HBV probability and human population density (persons/km^2^).

Despite this limitation, our findings provide new data and perspectives on the natural transmission of HBV from humans to multiple wild primate genera—an area that remains poorly explored. Rondônia, a heavily deforested Amazonian state, has undergone major demographic changes since the seventeenth century due to colonization and immigration. These influxes were driven by different development cycles of mining, slavery, rubber production, railway construction, hydroelectric development, and, more recently, international refugee settlement (de Oliveira & de Oliveira Amaral, 2018). Subsequently, these migration waves created a multicultural population, of which 57.7% are non-native (IBGE, 2015). Our phylogenetic analysis of five positive samples confirmed HBV-A and HBV-D genotypes, matching human strains prevalent in the region (Sitnik et al., 2004; Viana et al., 2005; Santos et al., 2010, Ferreira-Junior, 2020). This reflects the historic movement of tens of thousands of Africans and Europeans into this area. Initially, they were brought to help construct the Madeira–Mamore railway between 1907 and 1912, before the arrival of hundreds of thousands of Europeans to farm the land. Those strains match those found in our primate samples, which support the hypothesis of a human–primate transmission of HBV (Fig. 3).

**Figure 3:**
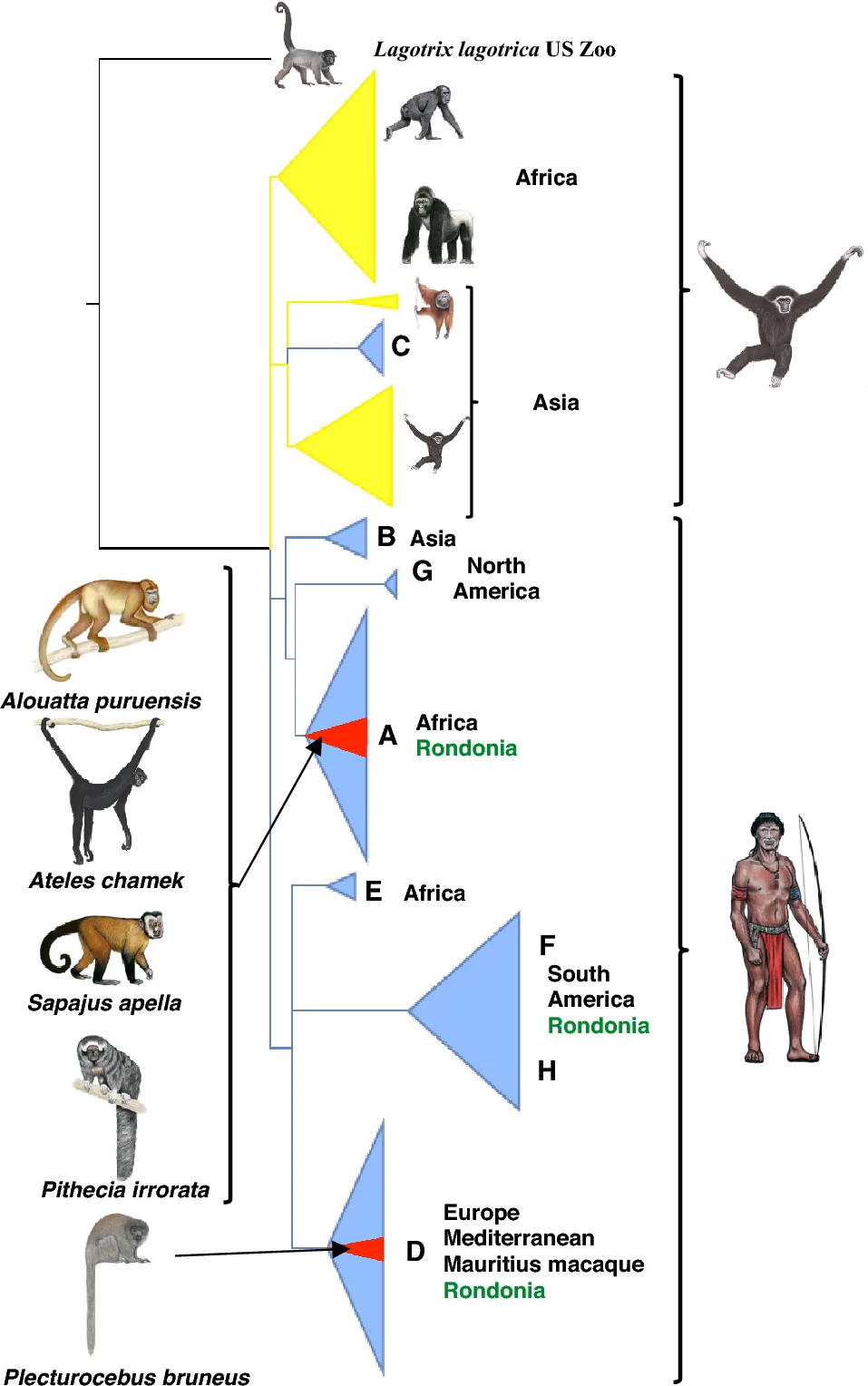
Bayesian tree based on a 547-bp fragment of the S gene for HBV different strains from humans (in blue), and African and Asian apes (in yellow). Our five New World primate samples from the southwestern Amazon are represented by red triangles within the collapsed clades. The human HBV types found in Rondônia are shown in green (Based on Supplementary Material, Fig. S2). Primate Illustrations copyright 2014 Stephen D. Nash/Conservation International/ IUCN SSC Primate Specialist group. Used with permission.

The absence of HBV in the remote Japurá site further supports the idea that human presence is the primary driver of HBV transmission to nonhuman primates. Similar findings have been reported from Mauritius, where an HBV strain that grouped with the human HBV genotype D was found in free-ranging macaques (*Macaca fascicularis*) (Dupinay et al., 2013). HBV has been detected in the saliva of HBV carriers (Noppornpanth et al., 2000; Mahboobi et al., 2012), and it is a proven transmission route between humans (Hui et al., 2005) and from humans to gibbons under laboratory conditions (Bancroft et al., 1977; Scott et al., 1980). An HBV strain isolated from a woolly monkey (*Lagothrix lagotricha*) at the Louisville Zoo was not related to any known HBV strain. Hu et al. (2000) reported that this strain was related to the human HBV-F strain, but our analysis (see supplementary materials, Fig. S2) supports an earlier study (Lanford et al., 1998), which suggested that this is the most differentiated and basal form among primate HBV strains. In the present study, both of our woolly monkey samples tested negative. While apes have developed species-specific HBV strains, only human HBV strains have been found in both Old and New World monkeys.

Deforestation and human encroachment into wildlife habitats increase human–primate interactions, facilitating cross-species disease transmission. HBV transmission can potentially be more likely to occur through hunting, wild meat consumption, and the pet trade. In the Amazon, an estimated 3 million individuals of three main primate genera (*Alouatta*, *Ateles* and *Sapajus*) are consumed annually for subsistence by Indigenous people and local communities and an estimated 1,500 kg of primate meat is consumed annually in five cities (Antunes et al., 2025; El Bizri et al., 2020). Many primate species, in particular squirrel monkeys (*Saimiri* spp.), are frequently traded as pets (D’Cruze et al., 2021). This raises concerns about the potential spillback of HBV or other pathogens into human populations (Fagre et al., 2022).

Experimental studies show that HBV-infected chimpanzees can develop symptoms such as anorexia, lethargy, and jaundice. The extent to which HBV infection has any clinical effect on New World primates remains to be investigated. The presence of human HBV genotypes in New World primates, coupled with the absence of HBV in less anthropogenetically affected areas, strongly supports the notion that humans are the source of HBV infections in wild Amazonian primates. Given the significant human–wildlife interaction in the region, there is a need for greater surveillance and preventive measures to mitigate the risk of disease transmission.

## Supplementary Information

Below is the link to the electronic supplementary material.

Supplementary file1 (DOC 149 KB)

Supplementary file2 (DOCX 1411 KB)
