# Supplementary material for: First Report of Human Hepatitis B Virus (HBV) in Wild Neotropical Primates
Source: Ecohealth. 2026 Apr 3;23(3):392–7. doi: 10.1007/s10393-026-01787-5 (PMC13582035; doi:10.1007/s10393-026-01787-5)
Supplement: Supplementary file 1 [file 10393_2026_1787_MOESM2_ESM.docx]

# Supplementary Materials

# Detailed Methodology

Primates were sampled in two separate sites of the Brazilian Amazon between July 2009 and November 2013: 1) in the Southwestern Amazonia (i.e., municipalities in the state of Rondônia and two nearby cities in Mato Grosso and Amazonas state), a region with high human occupation (i.e., 1,777 million inhabitants), urbanization, and deforestation (i.e., around 31% of its native vegetation lost in the last 30 years); and 2) the upper Japurá River region, a remote area deep in the Amazon that is mostly unaffected by humans. Animals were all wild and either captured live to obtain blood samples (Southwestern Amazonia: 49 samples of 17 species) or collected as voucher specimens to acquire liver tissues (Japurá: 39 samples of 11 species) (Table S1) and represented a wide range of species (Fig 1 A).

Details of the sampling procedures and protocols are described elsewhere (Araújo et al., 2013). The collection procedure was carried out in two ways. In Southwestern Amazonia, primates were captured alive, blood samples were collected, and individuals were released at their capture sites, as euthanasia was not permitted. In contrast, samples from the remote area were obtained through a separate, government-sponsored project aimed at surveying remote Amazonian regions and collecting voucher specimens for vertebrate collections in Brazilian institutions. Blood collection from animals in the remote area was not included in that protocol; therefore, only tissue samples (e.g., muscle, heart, and liver) were available for analysis. In both situations, blood and tissues were kept in a -80°C freezer. For blood samples, we extracted viral DNA from 200 microliters (μL) of serum using an *in-house* method based on membrane lysis with guanidine isothiocyanate and chloroform, adapted from a previously reported method (Chomczynski, 1993). DNA was extracted from all tissues using a standard phenol-chloroform protocol. (Sambrook et al., 2006). Following DNA extraction, amplification was performed by duplex polymerase chain reaction (PCR) to generate amplicons of 1.5 kilobases (kb) and 1.7 kb, as described by Tran et al. (2006). If these two bands were not detected by agarose gel electrophoresis, the amplicons were subjected to nested PCR amplification of a 741-base pair (bp) fragment of the rt/S gene using RT/S forward (5′-AAGGTATGTTGCCCCGTTTGTC-3′) and RT/S reverse (5′-GGGTTGCGTCAGCAAACACT-3′) primers, according to Gauthier et al. (2010). All PCR products were separated by agarose gel electrophoresis adding also HPV positive (from cultured cells) and negative controls and a blank sample (no DNA) that showed no amplification (Fig. S2). Positive PCR products were purified using an ExoSAP-IT kit (GE, USA) according to the manufacturer’s instructions, and then sequenced with an ABI Prism® 377 Automatic Sequencer (Applied Biosystems, Foster City, CA, USA).

All the sequences were confirmed by eye using ProSeq 3.0 (Filatov, 2002). A 547-bp fragment of the S gene was used in the final phylogenetic analysis, which included three additional datasets. The first comprised representative sequences for human HBV genotypes (Genebank accession numbers in brackets) A–H (**A:** V00866, X51970, X70185, S50225; **B:** U95551, X97848, X85254, X65257; **C:** D16665, D50489, D16666-7, D50517, D12980, D50519, AY123041, D50520, D00630, D23682, X52939, V00867, X04615, M38636, D23684, X01587, D28880, D23680-1; **D:** D00329-31, M54923, X97850-1, D23678-9, D50521-2; **E:** X75664, HE974384, X75657; **F:** HE981183, X75663, X75658, HE974368-9, X69798, **G:** HE981172-3, HE981175; **H:** AB516394-5, AB846650). The second comprised HBV sequences from gorillas (AJ131567, FJ798095-7, JQ664502-3), chimpanzees (D00220, AB032432-3, AF222322-3, AF242585-6, AF305327, AF498266, AM117395-7, AY330911-2, FJ798098-9, HQ018763-4, JQ664504-9), gibbons (AB037927-8, AY077735-6, AY330915-7, AY781184), orangutans (AF193863-4) and the woolly monkey (AF046996) from Louisville Zoo.

We employed a generalized linear mixed model (GLMM) to investigate the influence of human population density (persons/km²), deforestation level (percentage of native cover loss), and Gross Domestic Product per capita (GDP per capita, in US$) on the likelihood of primates testing positive for HBV at different sampling sites (Fig. 2). We treated HBV status as a binary response variable, coding positive samples as 0 and negative samples as 1, stratified by species and sampling site. The model was run using the *gamlss* package in R studio (Version 2023.09.1+494) with a Binomial distribution family. Our approach involved executing a stepwise regression procedure that incorporated both backward and forward selection methods. This stepwise process iteratively adds or removes explanatory variables based on their contribution to enhancing the model's fit. We incorporated primate species as a random factor to account for unmeasured species-specific effects, such as varying susceptibilities to HBV influenced by genetic, physiological, and behavioural factors. This provided a more accurate and generalizable understanding of the factors influencing HBV prevalence across diverse primate species. In addition, we also applied the 'weights' term to adjust for disparities in sample sizes across different primate species and collection sites, ensuring a more balanced and representative model outcome.

The selection of variables was done by the Akaike Information Criterion (AIC). The AIC not only considers the goodness of fit but also incorporates a penalty for the number of parameters in the model, thereby balancing model complexity against the risk of overfitting. In each step of the regression, the AIC of the model was calculated, and the variable whose addition or removal resulted in the lowest AIC was chosen for the next iteration. This process continued until no further reduction in AIC was possible, at which point we obtained our final model.

Finally, human strains and genotypes from patients in Rondônia (HM101096-100, HM101102, HM101104-12, HM101114-30) were compared directly with our primate samples to determine the genotypes of HBV strains infecting primates. Sequences were aligned using CLUSTALX 2.1 (Larkin et al., 2007). The most appropriate evolutionary model for this dataset was defined as GTR+I+G based on Akaike’s information criterion (Akaike, 1974) using MrModeltest 2.3 (Nylander, 2004). Bayesian analysis was performed using MrBayes 3.2 (Ronquist et al., 2012) for 10,000,000 generations with sampling every 100 generations, where the first 10% were discarded as the burn-in (Fig. S2).

Strict procedures were followed to avoid false positives (Kwok and Higuchi, 1989), including splitting the samples and performing extraction procedures at two different times. All procedures adopted in this study were approved, in full compliance with specific federal guidelines issued by the Brazilian Ministry of Environment (SISBIO, process numbers 14081–3 and 17302–1).

Figure S1. Agarose gel electrophoresis of PCR products for HPV detection. Lane M contains the DNA size marker (molecular weight ladder) used to estimate amplicon size. Samples S136, S143, S150, and S166 present amplification bands consistent with the presence of HPV DNA. Samples C+ are positive controls from a cultured HBV sample, exhibiting the expected HPV-specific bands. Samples C- and *C. branco* are negative controls (no DNA), with no amplification observed.

Figure S2. Bayesian tree based on a 547-bp fragment of the HBV S gene of different strains of the virus from Humans alongside Apes from Africa and Asia. Our five New World Primate samples from southwestern Amazonia are shown in red, and sequences from Human Rondônia patients are shown in green. Node values correspond to the posterior probability.

# References

Akaike H (1974) A new look at the statistical model identification. *IEEE Transactions on Automatic Control* 19:716-723.

Araújo MS, Messias MR, Figueiró MR, Gil LHS, Probst CM, Vidal NM, Katsuragawa TH, Krieger MA, Pereira da Silva LH, Ozaki LS (2013) Natural Plasmodium infection in monkeys in the state of Rondônia (Brazilian Western Amazon). *Malaria Journal* 12:1-8.

Chomczynski P (1993) A reagent for the single-step simultaneous isolation of RNA, DNA and proteins from cell and tissue samples. *Biotechniques* 15:532-534.

Filatov DA (2002) ProSeq: a software for preparation and evolutionary analysis of DNA sequence data sets. *Molecular Ecology Notes* 2:621-624.

Gauthier M, Bonnaud B, Arsac M, Lavocat F, Maisetti J, Kay A, Simon F, Zoulim F, Vernet G (2010) Microarray for hepatitis B virus genotyping and detection of 994 mutations along the genome. *Journal of Clinical Microbiology* 48:4207-4215.

Kwok SA, Higuchi R (1989) Avoiding false positives with PCR. *Nature* 339:237-238.

Larkin MA, Blackshields G, Brown NP, Chenna R, McGettigan PA, McWilliam H, Valentin F, Wallace IM, Wilm A, Lopez R, Thompson JD (2007) Clustal W and Clustal X version 2.0. *Bioinformatics* 23:2947-2948.

Nylander JAA (2004) MrModeltest v2. Program distributed by the author. Evolutionary Biology Centre, Uppsala University, Sweden.

Ronquist F, Teslenko M, Van Der Mark P, Ayres DL, Darling A, Höhna S, Larget B, Liu L, Suchard MA, Huelsenbeck JP (2012) MrBayes 3.2: efficient Bayesian phylogenetic inference and model choice across a large model space. *Systematic Biology* 61:539-542.

Sambrook JR, Russell DW, Sambrook J (2006) The condensed protocols from molecular cloning: a laboratory manual, New York, USA: Cold Spring Harbor Laboratory Press.

Tran N, Berne R, Chann R, Gauthier M, Martin D, Armand MA, Ollivet A, Teo CG, Ijaz S, Flichman D, Brunetto M (2006) European multicenter evaluation of high-density DNA probe arrays for detection of hepatitis B virus resistance mutations and identification of genotypes. *Journal of Clinical Microbiology* 44:2792-2800.
