# Supplementary material for: First Report of Human Hepatitis B Virus (HBV) in Wild Neotropical Primates
Source: Ecohealth. 2026 Apr 3;23(3):392–7. doi: 10.1007/s10393-026-01787-5 (PMC13582035; doi:10.1007/s10393-026-01787-5)
Supplement: Supplementary file 2 [file 10393_2026_1787_MOESM1_ESM.doc]

**Supplemental data**

**Table S1:** List of wild New World Primate samples from specimens caught in Rondônia and Japurá areas, Brazil; result of hepatitis B (reaction) in two different types of samples.

| **Species** | **Locality** | **Reaction** | **Latitude** | **Longitude** | **Sample** |
| --- | --- | --- | --- | --- | --- |
| *Alouatta caraya* | São Francisco do Guaporé | Negative | -12.50 | -63.58 | blood |
| *Alouatta puruensis* | Porto Velho | Negative | -9.29 | -64.65 | blood |
| *Alouatta puruensis* | Porto Velho | Negative | -9.27 | -64.71 | blood |
| *Alouatta puruensis* | Cabixi | Negative | -13.38 | -60.58 | blood |
| *Aotus nigriceps* | Pimenta Bueno | Negative | -12.08 | -60.62 | blood |
| *Aotus nigriceps* | Comodoro | Negative | -13.52 | -60.43 | blood |
| *Ateles chamek* | Porto Velho | Negative | -8.69 | -63.87 | blood |
| *Ateles chamek* | Pimenta Bueno | Negative | -12.06 | -60.68 | blood |
| *Ateles chamek* | Machadinho do Oeste | Negative | -8.60 | -62.40 | blood |
| *Callicebus brunneus* | Porto Velho | Negative | -12.06 | -60.68 | blood |
| *Callicebus brunneus* | Itapuã do Oeste | Negative | -8.75 | -62.98 | blood |
| *Callicebus brunneus* | Candeias do Jamari | Negative | -11.97 | -60.70 | blood |
| *Callicebus cinerascens* | Pimenta Bueno | Negative | -13.53 | -60.43 | blood |
| *Callicebus cinerascens* | Comodoro | Negative | -8.83 | -64.00 | blood |
| *Cebus albifrons* | Humaitá | Negative | -8.19 | -64.12 | blood |
| *Chiropotes albinasus* | Machadinho do Oeste | Negative | -8.66 | -62.38 | blood |
| *Lagothrix cana cana* | Machadinho do Oeste | Negative | -8.64 | -62.37 | blood |
| *Lagothrix cana cana* | Comodoro | Negative | -13.50 | -60.29 | blood |
| *Mico melanurus* | Pimenta Bueno | Negative | -12.06 | -60.62 | blood |
| *Pithecia irrorata* | Porto Velho | Negative | -9.29 | -64.62 | blood |
| *Pithecia irrorata* | Pimenta Bueno | Negative | -12.08 | -60.62 | blood |
| *Pithecia irrorata* | Porto Velho | Negative | -9.30 | -64.67 | blood |
| *Pithecia irrorata* | Porto Velho | Negative | -9.26 | -64.62 | blood |
| *Pithecia irrorata* | Humaitá | Negative | -8.23 | -64.02 | blood |
| *Pithecia irrorata* | São Francisco do Guaporé | Negative | -12.50 | -63.58 | blood |
| *Pithecia irrorata* | São Francisco do Guaporé | Negative | -12.50 | -63.58 | blood |
| *Saguinus fuscicollis* | Itapuã do Oeste | Negative | -12.12 | -60.63 | blood |
| *Saguinus fuscicollis* | Candeias do Jamari | Negative | -8.84 | -63.69 | blood |
| *Saguinus fuscicollis* | Candeias do Jamari | Negative | -8.83 | -63.69 | blood |
| *Saimiri ustus* | Cujubim | Negative | -8.79 | -62.67 | blood |
| *Saimiri ustus* | Cabixi | Negative | -13.42 | -60.54 | blood |
| *Sapajus apella* | São Francisco do Guaporé | Negative | -12.50 | -63.58 | blood |
| *Alouatta puruensis* | Cabixi | Positive | -13.40 | -60.58 | blood |
| *Aotus nigriceps* | Porto Velho | Positive | -9.05 | -64.27 | blood |
| *Ateles chamek* | Porto Velho | Positive | -9.88 | -62.80 | blood |
| *Ateles chamek* | Comodoro | Positive | -13.53 | -60.43 | blood |
| *Ateles chamek* | São Francisco do Guaporé | Positive | -12.50 | -63.58 | blood |
| *Callicebus bernhardi* | Machadinho do Oeste | Positive | -8.60 | -62.41 | blood |
| *Callicebus brunneus* | Candeias do Jamari | Positive | -8.75 | -62.98 | blood |
| *Callicebus cinerascens* | Comodoro | Positive | -13.53 | -60.44 | blood |
| *Cebuella pygmaea niveiventris* | Porto Velho | Positive | -8.82 | -63.99 | blood |
| *Pithecia irrorata* | Porto Velho | Positive | -9.27 | -64.63 | blood |
| *Pithecia irrorata* | Porto Velho | Positive | -8.88 | -64.03 | blood |
| *Pithecia irrorata* | Porto Velho | Positive | -8.83 | -63.94 | blood |
| *Pithecia irrorata* | Porto Velho | Positive | -9.59 | -64.99 | blood |
| *Saguinus labiatus* | Humaitá | Positive | -8.16 | -64.05 | blood |
| *Saimiri ustus* | Humaitá | Positive | -8.23 | -64.02 | blood |
| *Sapajus apella* | Machadinho do Oeste | Positive | -8.67 | -62.37 | blood |
| *Sapajus apella* | Humaitá | Positive | -8.19 | -64.05 | blood |
| *Alouatta seniculus* | Middle Japurá river | negative | -1.85 | -69.05 | liver |
| *Alouatta seniculus* | Middle Japurá river | negative | -1.84 | -69.02 | liver |
| *Alouatta seniculus* | Middle Japurá river | negative | -1.84 | -69.02 | liver |
| *Alouatta seniculus* | Middle Japurá river | negative | -1.70 | -69.17 | liver |
| *Alouatta seniculus* | Middle Japurá river | negative | -1.70 | -69.17 | liver |
| *Aotus trivirgatus* | Middle Japurá river | negative | -1.66 | -69.20 | liver |
| *Cacajao melanocephalus* | Middle Japurá river | negative | -1.64 | -69.20 | liver |
| *Cacajao melanocephalus* | Middle Japurá river | negative | -1.64 | -69.20 | liver |
| *Cacajao melanocephalus* | Middle Japurá river | negative | -1.64 | -69.20 | liver |
| *Callicebus torquatus* | Middle Japurá river | negative | -1.84 | -69.03 | liver |
| *Callicebus torquatus* | Middle Japurá river | negative | -1.87 | -69.05 | liver |
| *Callicebus torquatus* | Middle Japurá river | negative | -1.87 | -69.05 | liver |
| *Callicebus torquatus* | Middle Japurá river | negative | -1.71 | -69.11 | liver |
| *Callicebus torquatus* | Middle Japurá river | negative | -1.71 | -69.11 | liver |
| *Callicebus torquatus* | Middle Japurá river | negative | -1.70 | -69.13 | liver |
| *Cebuella pygmaea pygmaea* | Middle Japurá river | negative | -1.84 | -69.02 | liver |
| *Cebuella pygmaea pygmaea* | Middle Japurá river | negative | -1.84 | -69.02 | liver |
| *Cebuella pygmaea pygmaea* | Middle Japurá river | negative | -1.84 | -69.02 | liver |
| *Cebus unicolor* | Middle Japurá river | negative | -1.71 | -69.11 | liver |
| *Cebus unicolor* | Middle Japurá river | negative | -1.71 | -69.11 | liver |
| *Cebus unicolor* | Middle Japurá river | negative | -1.71 | -69.11 | liver |
| *Cebus unicolor* | Middle Japurá river | negative | -1.71 | -69.11 | liver |
| *Lagothrix lagothrica* | Middle Japurá river | negative | -1.86 | -69.38 | liver |
| *Lagothrix lagothrica* | Middle Japurá river | negative | -1.84 | -69.03 | liver |
| *Lagothrix lagothrica* | Middle Japurá river | negative | -1.87 | -69.04 | liver |
| *Pithecia hirsuta* | Middle Japurá river | negative | -1.85 | -69.05 | liver |
| *Pithecia hirsuta* | Middle Japurá river | negative | -1.86 | -69.03 | liver |
| *Pithecia hirsuta* | Middle Japurá river | negative | -1.86 | -69.03 | liver |
| *Saguinus fuscicollis* | Middle Japurá river | negative | -1.87 | -69.04 | liver |
| *Saguinus fuscicollis* | Middle Japurá river | negative | -1.87 | -69.04 | liver |
| *Saimiri cassiquiarensis* | Middle Japurá river | negative | -1.64 | -69.20 | liver |
| *Saimiri cassiquiarensis* | Middle Japurá river | negative | -1.67 | -69.28 | liver |
| *Saimiri cassiquiarensis* | Middle Japurá river | negative | -1.67 | -69.28 | liver |
| *Saimiri cassiquiarensis* | Middle Japurá river | negative | -1.84 | -69.03 | liver |
| *Saimiri cassiquiarensis* | Middle Japurá river | negative | -1.84 | -69.03 | liver |
| *Sapajus macrocephalus* | Middle Japurá river | negative | -1.72 | -69.12 | liver |
| *Sapajus macrocephalus* | Middle Japurá river | negative | -1.86 | -69.03 | liver |
| *Sapajus macrocephalus* | Middle Japurá river | negative | -1.85 | -69.05 | liver |
| *Sapajus macrocephalus* | Middle Japurá river | negative | -1.84 | -69.03 | liver |
